# Radiosensitization of Human Leukemic HL-60 Cells by ATR Kinase Inhibitor (VE-821): Phosphoproteomic Analysis

**DOI:** 10.3390/ijms150712007

**Published:** 2014-07-07

**Authors:** Barbora Šalovská, Ivo Fabrik, Kamila Ďurišová, Marek Link, Jiřina Vávrová, Martina Řezáčová, Aleš Tichý

**Affiliations:** 1 Institute of Medical Biochemistry, Faculty of Medicine in Hradec Králové, Charles University in Prague, Hradec Kralove 500 00, Czech Republic; E-Mails: salob5aa@lfhk.cuni.cz (B.S.); RezacovaM@lfhk.cuni.cz (M.R.); 2 Institute of Molecular Pathology, Faculty of Health Sciences in Hradec Králové, University of Defense in Brno, Hradec Kralove 500 01, Czech Republic; E-Mails: fabrik@pmfhk.cz (I.F.); link@pmfhk.cz (M.L.); 3 Department of Radiobiology, Faculty of Health Sciences in Hradec Králové, University of Defense in Brno, Hradec Kralove 500 01, Czech Republic; E-Mails: durisovak@pmfhk.cz (K.D.); vavrova@pmfhk.cz (J.V.)

**Keywords:** small-molecule kinase inhibitors, VE-821, ATR kinase, DNA damage response, radio-sensitization, quantitative phosphoproteomics, titanium dioxide chromatography, SILAC, leukemia, HL-60 cells

## Abstract

DNA damaging agents such as ionizing radiation or chemotherapy are frequently used in oncology. DNA damage response (DDR)—triggered by radiation-induced double strand breaks—is orchestrated mainly by three Phosphatidylinositol 3-kinase-related kinases (PIKKs): Ataxia teleangiectasia mutated (ATM), DNA-dependent protein kinase (DNA-PK) and ATM and Rad3-related kinase (ATR). Their activation promotes cell-cycle arrest and facilitates DNA damage repair, resulting in radioresistance. Recently developed specific ATR inhibitor, VE-821 (3-amino-6-(4-(methylsulfonyl)phenyl)-*N*-phenylpyrazine-2-carboxamide), has been reported to have a significant radio- and chemo-sensitizing effect delimited to cancer cells (largely p53-deficient) without affecting normal cells. In this study, we employed SILAC-based quantitative phosphoproteomics to describe the mechanism of the radiosensitizing effect of VE-821 in human promyelocytic leukemic cells HL-60 (p53-negative). Hydrophilic interaction liquid chromatography (HILIC)-prefractionation with TiO_2_-enrichment and nano-liquid chromatography—tandem mass spectrometry (LC-MS/MS) analysis revealed 9834 phosphorylation sites. Proteins with differentially up-/down*-*regulated phosphorylation were mostly localized in the nucleus and were involved in cellular processes such as DDR, all phases of the cell cycle, and cell division. Moreover, sequence motif analysis revealed significant changes in the activities of kinases involved in these processes. Taken together, our data indicates that ATR kinase has multiple roles in response to DNA damage throughout the cell cycle and that its inhibitor VE-821 is a potent radiosensitizing agent for p53-negative HL-60 cells.

## 1. Introduction

One of the treatment modalities in oncology is radiotherapy. It often employs chemical agents increasing sensitivity towards ionizing radiation (IR). IR induces the most deleterious lesions of DNA, double strand breaks (DSB), and their repair is regulated by ataxia telangiectasia-mutated kinase (ATM), DNA-dependent protein kinase (DNA-PK), and ATM and Rad3-related kinase (ATR). While activation of ATM and DNA-PK is triggered by DNA double stranded breaks*,* ATR kinase responds to a broad spectrum of agents inducing single stranded DNA [1]. ATR acts primarily in S*-* and G2-phases and responds to replication and genotoxic stress, however, a recent report has shown that ATR is activated also in irradiated G1 phase cells [2].

In 2011, a specific inhibitor of ATR, VE-821 (3-amino-6-(4-(methylsulfonyl)phenyl)-*N*-phenylpyrazine-2-carboxamide), was developed [3]. Its excellent selectivity in regards to sensitization of cancer cells towards various types of DNA damaging agents leaving healthy cells unaffected has been recently reported [4,5,6,7]. VE-821 selectivity is based on the concept that (i) more than 50% of cancer cells have lost their G1-phase checkpoint for example due to p53 mutation/deletion and thus rely on S- and G2-checkpoints; which are known to be regulated by ATR and (ii) cancer cells with activated oncogenes generate replication stress at much higher levels than normal cells, thus activating ATR [8,9]. This hypothesis is based on well-established “classical” methodology in molecular biology comprising western-blotting, confocal microscopy, flow-cytometry and so forth. However, this approach is often focused on one or two particular mechanisms not reflecting the complexity of cellular signaling pathways. Therefore it fails to give a comprehensive view of the mechanisms of radiosensitization by ATR inhibition in the context of cancer cells.

In the DNA damage response as well as in the other molecular processes (such as transcriptional and translational regulation, proliferation, differentiation, apoptosis, cell survival and many others) phosphorylation frequently initiates and propagates signal transduction pathways [10]. Therefore we decided to apply a phosphoproteomic approach in order to study the effect of specific inhibition of ATR by the small molecule inhibitor VE-821.

Our goal was to describe the changes in the phosphoproteome in radiosensitized tumor cells lacking functional protein p53. Although mass spectrometry of phosphopeptides obtained from tryptic protein digests has become a powerful tool for characterization of phosphoproteins involved in cellular processes, there is an inevitable part of the protocol: phosphopeptide enrichment. It compensates the low abundance, insufficient ionization, and suppression effects of non-phosphorylated peptides [11]. Hence, we optimized the metal oxide affinity chromatography (MOAC) enrichment using titanium dioxide and employed it in our experiments [12].

We have previously compared the effects of inhibitors of ATM (KU55933) and ATR kinases (VE-821) on the radiosensitization of human promyelocytic leukemia cells (HL-60), lacking functional protein p53. The inhibition of ATR by its specific inhibitor VE-821 resulted in a more pronounced radiosensitizing effect in HL-60 cells compared to the inhibition of ATM. In contrast to KU55933, the VE-821 treatment prevented HL-60 cells from undergoing G2 cell cycle arrest [13].

In this paper we characterize the radiosensitizing effect of VE-821 from a new perspective and we report the mechanisms and signaling pathways involved in the processes triggered by IR in p53-negative leukemic cells.

## 2. Results

Our goal was to describe the changes in the phosphoproteome in radiosensitized tumor cells, since in the DNA damage response (as well as in other molecular processes) phosphorylation frequently initiates and propagates signal transduction pathways. We aimed to characterize the effect of specific inhibition of ATR kinase by VE-821 and to report the mechanisms and signaling pathways involved in the processes triggered by IR in p53-negative human promyelocytic leukemic cells HL-60 that are ATR-dependent. To reach our goal, we employed SILAC-based quantitative phosphoproteomics together with metal oxide affinity chromatography using TiO_2_ microparticles to specifically enrich for phosphorylated peptides. To increase the number of identified and quantified phosphorylation sites, peptides were prefractionated by hydrophilic interaction liquid chromatography (HILIC) chromatography prior to the enrichment. Additionally, to make sure that the changes in the phosphoproteome are not based on changes of protein abundance caused by alterations in gene expression or protein degradation that could occur because of the relatively long incubation time (1 h after irradiation, and 1.5 h after VE-821 treatment), we also analyzed a non-phosphorylated complement of phosphorylated peptides obtained from flow-through fractions of the phosphopeptide enrichment.

### 2.1. Overall Phosphoproteomic Analysis Reveals Thousands of Phosphorylation Sites

In summary, we identified 9834 phosphorylation sites from 3210 protein groups, among them 4809 were quantified in all three experimental replicates. The phosphosite ratios were shown to correlate well between the replicates (Pearson correlation coefficient was between 0.8 and 0.87). Moreover, the correlation between the normalized *H*/*L* ratios of phosphopeptides and the normalized protein *H*/*L* ratios was also calculated (the calculation was based on 1941 phosphorylation sites from 557 proteins), and the low correlation coefficient value (*R* = 0.0162) indicated that the quantified changes in the phosphoproteome were presumably not caused by changes in the protein abundance. The overview of all identified phosphorylation sites is given in the supplementary (Table S1).

To determine whether a phosphorylation site exhibits an appropriate alteration in abundance in all of the replicates to be considered as differentially regulated after the treatment, we employed the Global rank test (GRT) with the non-parametric estimate of the false discovery rate (FDR; see material and methods). At the 1% FDR level, 336 phosphorylation sites (260 proteins) were evaluated as differentially up-regulated, whereas 202 phosphorylation sites (168 proteins) were differentially down-regulated (some of the proteins contained both down*-* and up-regulated phosphorylation sites). Since a kinase inhibitor was applied to modulate the response to IR induced DNA damage, the number of down-regulated sites would be expected to be larger than the number of up-regulated sites. However, the number of up-regulated sites increased and possibly reflected relatively long time period that passed from the initial treatment, and allowed the cells to eventually trigger a compensatory phosphorylation response in order to overcome the inhibitory effect of VE-821.

### 2.2. Combination of Ionizing Radiation and VE-821 Treatment Abrogated Phosphorylation of Checkpoint Kinase-1 on Ser345

**Figure 1:**
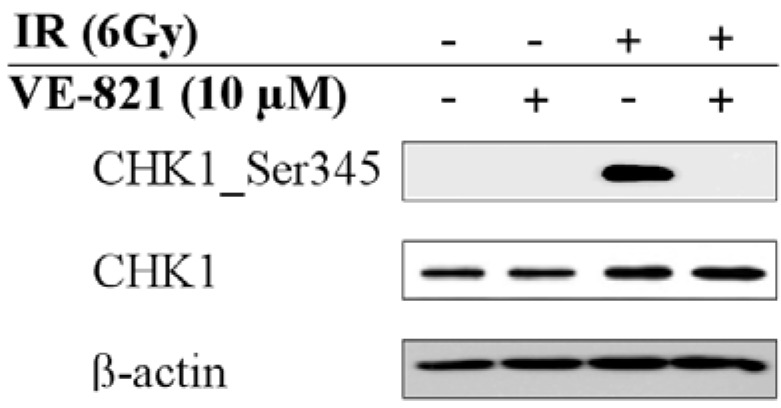
In order to confirm the inhibitory effect of VE-821 on ATM and Rad3-related kinase (ATR) we assessed phosphorylation of CHK1 on Ser345 (a direct downstream target of ATR) and we found this particular phosphorylation to be inhibited. Beta-actin was used as a gel loading control. The representative blots from at least three independent experiments are shown.

In order to confirm the inhibitory effect of VE-821 on ATR in our model, we assessed phosphorylation of checkpoint kinase-1 (CHK1) on Ser345. Since it reports active and functional ATR, this particular direct downstream target of ATR was found to be phosphorylated upon irradiation. As shown in Figure 1, CHK1 phosphorylation was abrogated after VE-821 pre-incubation.

### 2.3. Most of the Regulated Phosphoproteins Were Localized in the Nucleus and Related to Mitosis, Cell Cycle Regulation, DNA Damage Response and Gene Expression

**Figure 2:**
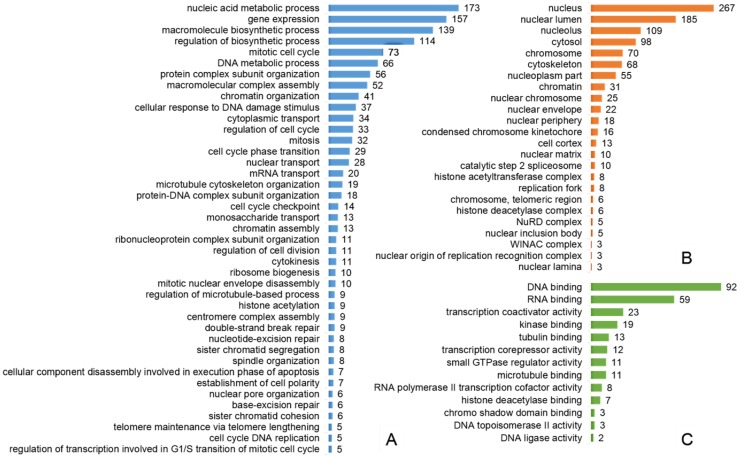
Selected results of Gene Ontology terms over-representation analysis (FDR < 0.01): (**A**) Overrepresented Gene Ontology (GO) Biological process level 4 terms; (**B**) Overrepresented GO Cellular component level 4 terms; (**C**) Overrepresented GO Molecular function level 4 terms. *x*-axis contains the number of proteins involved in a particular pathway that were found differentially phosphorylated in our study.

To functionally and spatially interpret the list of 396 phosphorylated proteins resulting from the GRT, Gene Ontology (GO) annotation was performed using the ConsensusPathDB, over-representation analysis “web tool” [14,15] and the summary of the results is given in Figure 2. The analysis of cellular component GO terms (level 4) revealed that most of the proteins were localized in the nucleus, many of them included in chromatin, localized at the centromeric region of a chromosome, replication fork or as a part of histone acetyltransferase or deacetylase complexes. Nevertheless, there was also a considerable fraction of proteins annotated to be a part of cytosol. The most over-represented molecular functions (level 4) of regulated phosphoproteins were binding of both nucleic acids (RNA binding and DNA binding), transcriptional co-activator and co-repressor activities, as well as DNA ligase or topoisomerase II activities. The list of overrepresented biological process terms (level 4) contained processes related to mitosis (nuclear envelope disassembly, sister chromatid cohesion, spindle localization or cytokinesis), cell cycle regulation (cell cycle progression, cell cycle phase transition, cell cycle checkpoint, regulation of transcription involved in G1/S transition of the mitotic cell cycle, cell cycle DNA replication), cellular response to DNA damage stimulus (base*-* and nucleotide*-*excision repair, double-strand break repair) or gene expression (mRNA transport, histone acetylation).

### 2.4. The Pathways Affected by Co-Treatment Were Involved in Cell Cycle Progression, Stimulus-Based Changes in Gene Expression, DDR and Apoptosis

**Figure 3:**
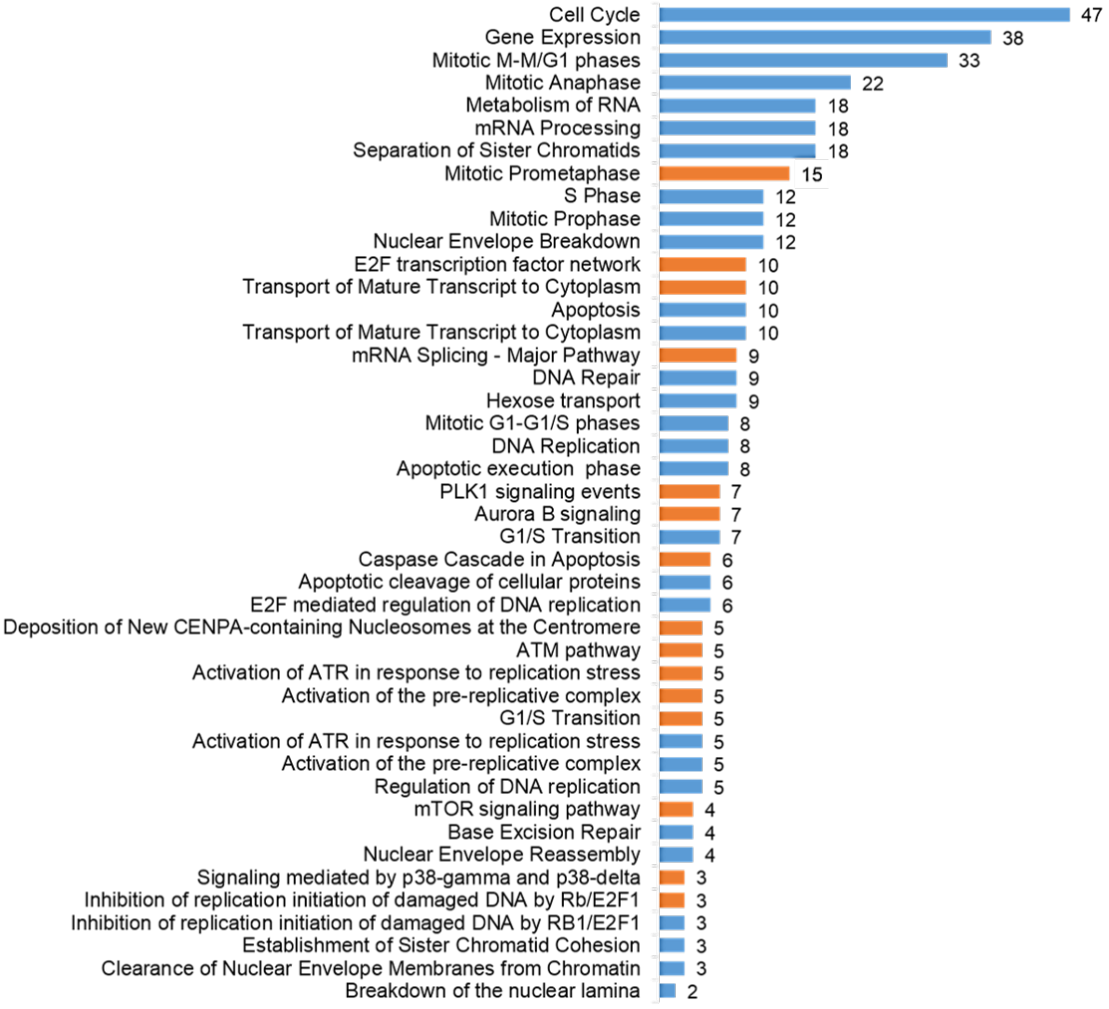
Selected results of Reactome pathways (blue bars) and Pathway Interaction Database (PID) pathways (orange bars) over-representation analysis (FDR < 0.01): *x*-axis shows the number of proteins involved in particular pathways that were differentially phosphorylated in our study.

To assess which signaling pathways contained differentially phosphorylated proteins and thus could be considered as triggered or modulated by ATR inhibition, we performed a pathway over-representation analysis using the same web-tool as in the case of GO enrichment, however, we addressed the signaling pathways stored in the Reactome pathway database [16] and Pathway Interaction Database (PID; [17]). The overrepresented pathways are shown in Figure 3. It is apparent that most of the pathways were related to different phases of the cell cycle and transitions between them, stimulus-based changes in gene expression, DNA damage repair and DNA damage-induced programmed cell death.

### 2.5. Significantly Down-Regulated Phosphorylation Sites Comprised SP/TP Motifs, while SQ/TQ Were amongst Up-Regulated Ones

**Figure 4:**
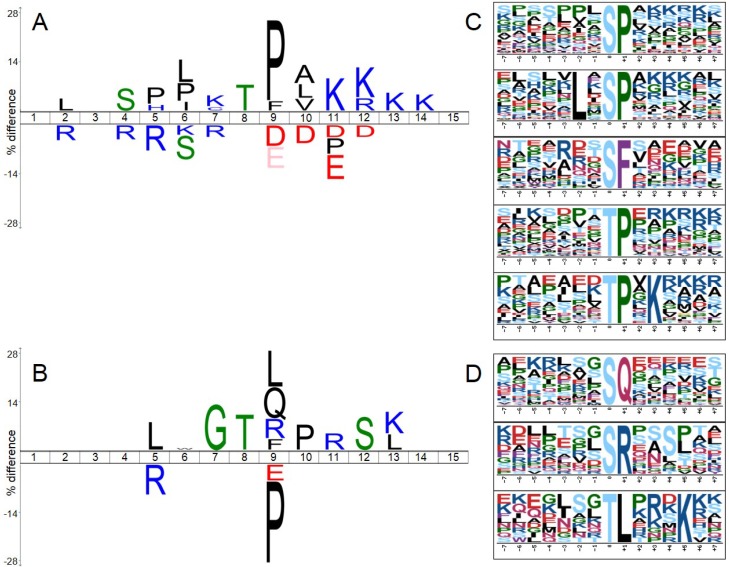
Sequence motifs analyses and visualization performed using iceLogo tool (**A**,**B**) and motif-x algorithm (**C**,**D**). The amino acid sequences of significantly differentially up- or down-regulated phosphorylation sites (**A**,**C** or **B**,**D** respectively) were analyzed against a statistical background comprising all class I sites revealed in our study. In **A** and **B**, amino acids that were more frequently observed in the proximity of a regulated phosphorylation site are indicated over the middle line, whereas the amino acids with lower frequency are indicated below the line; phosphorylated amino acid is located at position 8; **C** and **D** depict sequence motifs extracted by motif-x algorithm at a significance level of *p* < 0.00003 (which approximately corresponds to a *q*-value of 0.01 after Bonferroni correction for multiple hypothesis testing).

To analyze and visualize sequence motifs that were enriched among significantly regulated class I phosphorylation sites (see material and methods), we utilized the iceLogo tool [18] and the motif-x algorithm [19,20]. Amino acid sequences surrounding either up*-* or down*-*regulated phosphorylation sites were tested against a background reference set composed of sequences surrounding all phosphorylation sites revealed in our study that reached the minimal localization probability 0.75. Results of these analyses are shown in Figure 4.

Figure 4A,C depicts that among up-regulated sites the proline-directed and basic motifs were overrepresented whereas acidic motifs were significantly underrepresented (typical e.g., for casein kinases). SP/TP motifs followed by basic amino acids are known to be typical consensus motifs of cyclin-dependent kinases (CDKs), mitogen-activated protein kinases (MAPKs) or glycogen synthase kinase 3 (GSK-3). On the other hand, SP/TP motifs were underrepresented amongst significantly down-regulated phosphorylation sites (Figure 4B,D) while SQ/TQ motifs (typical for DNA repair enzymes), TL, SR, and GT motifs were significantly overrepresented.

### 2.6. Kinase Activity Analyses Predicts Various Kinase Substrates to Be Regulated

**Figure 5:**
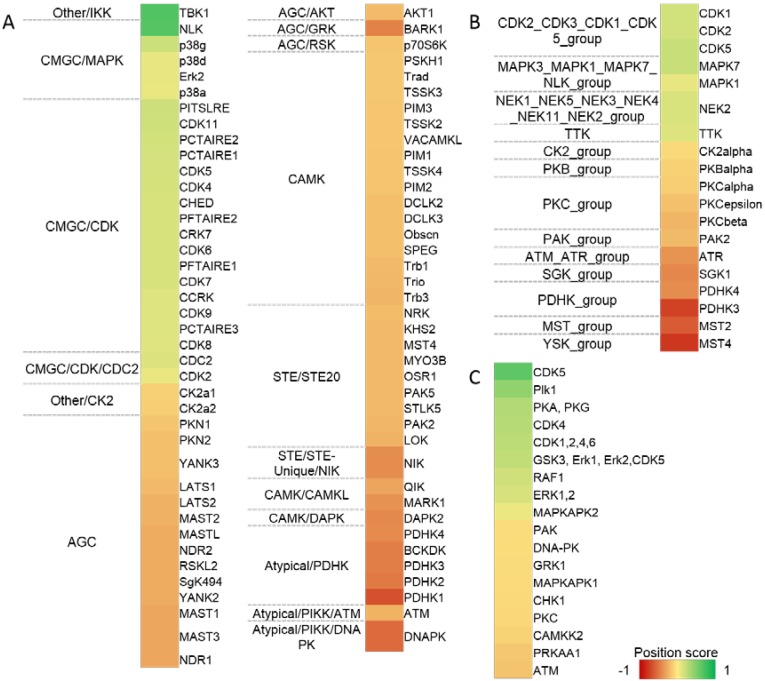
Analysis of kinases activity. Kinase-substrate relations predicted by iGPS (**A**) NetworKIN 3; and (**B**) linear motifs analysis derived from motifs stored in Human proteome reference database (HPRD) database; (**C**) The color shade corresponds to the value of the “position score” that indicates if the ratios (normalized *H*/*L* ratios) of sites phosphorylated by a particular kinase tend to be larger (0 to 1) or lower (−1 to 0) than the rest of the data (*p* < 0.05), *i.e.*, green color of a region in a heatmap indicates an increased activity of a particular kinase after IR and VE‑821 co-treatment, red color indicates trend of decreased activity.

There are several tools available to predict kinase-specific phosphorylation sites. Since each suffers its own limitations, the employment of different prediction algorithms seems to be a profitable approach to describe the alterations of intracellular signaling. Based on this assumption, at first we applied a very common approach relying on simple assigning of consensus motifs downloaded from HPRD (Human proteome reference database) to phosphorylated sequences. Consecutively, we employed two kinase predictors that apply consensus motif scoring together with the network context of kinases and their potential substrate, *i.e.*, NetworKIN 3 [21] and iGPS [22], differing in their motif scoring algorithms. The results are depicted in Figure 5. Only those kinases that reached the significance level of *p* < 0.05 are reported. The position score indicates the degree of regulation.

Our analyses confirmed the decrease in activity of the group of enzymes sharing the SQ/TQ motif (*i.e.*, ATM, ATR and DNA-PK). The remaining down-regulated kinases composed a quite heterogenic group of protein kinases including Casein kinase 2 (CK2), Protein kinase B (AKT) a subgroup of Protein kinases C (PKCs) or various isoforms of Pyruvate dehydrogenase kinase (PDK).

On the contrary, the increase in kinases with SP/TP specificity (*i.e.*, CDKSs, MAPKs, and GSK-3 *etc*.) was revealed. Additional groups of kinases that were indicated to be up-regulated mostly comprised kinases that were involved in mitosis, e.g., Polo-like kinase 1 (PLK1), Never in mitosis A-related kinase 2 (NEK2) or Dual specificity protein kinase TTK (TTK).

## 3. Discussion

DNA damage response (DDR) is an essential mechanism to maintain the genome integrity of cells. Moreover, its functionality implicates the efficiency of radio*-* or chemotherapeutic agents administered during cancer therapy and thus underlies cell resistance to genotoxic stress in normal as well as in cancer cells. Therefore, the therapeutic inhibition of DNA repair enzymes represents a promising strategy how to increase sensitivity towards genotoxic insults in cancer cells. One of the repair enzymes is the ATR kinase that is considered to primarily act in the S and G2 phases of the cell cycle and suppress the “physiological” replication stress as well as genotoxic stress induced by agents such as hypoxia, UV*-* and ionizing radiation, and hydroxyurea (HU) [8].

A recent discovery of the first selective inhibitors of ATR kinase facilitated investigation of the cellular functions of ATR and launched the development of new potential anti-cancer drugs targeting this kinase to sensitize cancer cells to chemo*-* or radiotherapy [3]. Reaper *et al*. confirmed that ATR inhibition using VE-821 that belongs to the group of 3-amino-6-pyrazines may offer great promise in cancer treatment. In concordance with previous results suggesting that disruption in ATM-p53 pathway should enhance the sensitivity of cells to the disruption of the ATR pathway [23], VE-821 was shown to selectively induce cytotoxicity in cancer cells without affecting normal cells. Treatment with 20 μM VE-821 alone induced irreversible growth arrest and apoptosis in a large fraction of different cancer cell lines that were p53-deficient (human colorectal carcinoma cell lines HT23, HT29 or human malignant melanoma cell line HT144) or p53*-*proficient but with a defective ATM-mediated pathway (human colorectal carcinoma cells HCT116). On the other hand, growth arrest induced by VE-821 in normal human lung fibroblasts (HFL-1) was fully reversible with minimal death [4].

In our previous study, we have confirmed the radiosensitizing effects of VE-821 in relatively radioresistant p53 negative cells of promyelocytic leukemia (HL-60). The inhibition of ATR (10 μM VE-821) in combination with IR (3 Gy) resulted in pronounced decreases in clonogenic survival and significant changes in the proportions of cells in S and G2 phases of the cell cycle as compared to irradiated control cells [13]. In our recent work, we employed the phosphoproteomic approach to describe the changes in intracellular signaling caused by radiosensitization in more detail. Our goal was to characterize signaling pathways triggered by irradiation and modulated by inhibition of an essential DNA repair enzyme ATR kinase. We aimed to reveal which kinases showed alterations in their activities in the presence of the ATR inhibitor VE-821 and thus were probably functionally linked to ATR-mediated response upon genotoxic stress. Moreover, deeper insight into processes triggered in cancer cells sensitive to ATR inhibition may in the future lead to identification of potential markers of sensitivity to VE-821 treatment.

Both “motif-network” kinase analyses confirmed an expected decrease in the activity of the ATR kinase that was inhibited in order to radiosensitize the HL-60 cells. However, a decrease in ATM kinase activity was also evaluated to be statistically significant. Since it was reported, that the activity of the ATM kinase is not affected by VE-821 in 10 μM concentration ([4,13]; assessed by monitoring of CHK2 Thr68 phosphorylation which is a widely accepted specific marker of ATM activity), we account it to the decrease in phosphorylation of the consensus SQ/TQ motif which is shared between ATR, ATM and also DNA-PK. Additionally, ATM and ATR kinases share overlapping substrate specificity and hence the addition of protein-protein interaction scoring into the analysis could not improve the scoring algorithm to further distinguish between them. Interestingly, the activity of CHK1, which is a direct downstream target of ATR, was revealed to be significantly decreased in the HPRD motifs analysis (*p*-value lower than 0.05), however the activity shift was evaluated to be lower than expected after ATR/CHK1 pathway disruption (position score of only −0.07, where position score of 0 indicates non-regulated activity and −1 strongly downshifted activity). Moreover, Ser296 autophosphorylation of CHK1 and importantly Ser216 phosphorylation of CDC25C (which is an accepted marker of functional CHK1 dependent signaling) were not significantly altered in our work. On the other hand, we detected down-regulation of phosphorylation at Ser345 by immunoblotting, which serves to localize CHK1 to the nucleus following checkpoint activation [24]. Another important CHK1 phosphorylation at Ser317 was not detected in our phosphoproteomic analysis. Altogether, these findings underline the important concepts that have been reported recently: (i) the abrogation of different phosphorylation sites at CHK1—and thus the ATR/CHK1 pathway*-*by VE-821—is cell line—and treatment-dependent [7]; (ii) the radiosensitizing mechanism of VE-821 cannot be attributed merely to the abrogation of the ATR/CHK1 pathway [2,7].

All analyses further confirmed an increase in the activity of CDKs (SP/TP consensus motifs) that are inhibited by DNA repair enzymes in response to genotoxic stress in order to induce the cell cycle arrest to allow the repair of the damaged DNA lesions. It has been reported previously, that p53-negative HL-60 cells irradiated by lower doses of IR (up to 10 Gy) cannot undergo the p53-dependent G1 cell cycle arrest and therefore transiently accumulate in S phase, arrest at G2/M cell cycle checkpoint and eventually die by a so-called postmitotic apoptosis [24]. In the presence of VE-821, the CDKs inhibitory effect of ATR kinase (that is normally activated by replication and genotoxic stress in S and the G2 phase of the cell cycle) diminished, which was proven by the cell cycle analysis; the proportion of cells in G2 phase significantly decreased when compared to irradiated cells indicating that this cell population proceeded into mitosis [13]. Together, these anticipated trends in ATR and CDKs activities revealed by bioinformatic processing of our data, proved their biological reliability and relevance in addition to calculated statistical significance.

IR induces various types of DNA damage in irradiated cells including base damage, DNA crosslinks, single strand breaks (SSBs) and importantly, the most deleterious double strand breaks (DSBs). While ATM is activated exclusively in response to DSBs, ATR responds to DSBs as well as to SSBs [25], however the response of ATR to DSBs that are not part of the replication process is ATM-dependent [26]. GO biological process (GOBP) over-representation analysis revealed a significantly overrepresented term “cellular response to DNA damage stimulus” that included 37 proteins with significantly changed phosphorylation measured in our experiments. Moreover, GOBP terms such as “base-excision repair”, “nucleotide-excision repair” or “double-strand break repair” were also significantly enriched. Overrepresented pathways comprised for example “Activation of ATR in response to replication stress” from Reactome database or “ATM pathway” from PID.

Naturally, the most desirable effect of ATR inhibition in cancer treatment is increased cell death. Our data indicated, that phosphorylation changes in signaling pathways involved in apoptosis were apparent 1 h after irradiation, which can be illustrated by results of pathways overrepresentation analysis (e.g., “Apoptosis”, “Apoptotic cleavage of cellular proteins”). Again, increased apoptosis induction in HL-60 cells after combined treatment with IR (3 Gy) and VE-821 has been reported previously [13].

The disruption of the G2/M cell cycle checkpoint by VE-821 can be monitored by the cell cycle analysis conducted by flow-cytometry 24 h after irradiation as a significant decrease of G2 phase cells in comparison to a control group of cells irradiated without VE-821 and was published in our previous work [13]. In our recent experiment, we observed multiple over-represented GOBP level 4 terms related to mitotic processes (e.g., “mitosis”, “centromere complex assembly”, “cytokinesis”, “sister chromatid cohesion”, or “spindle localization”) together with GO Cellular Component (GOCC) terms revealing the localization of some of the phosphorylated proteins at centromeric regions of condensed chromosomes (“condensed chromosome kinetochore”) which occur during mitosis. Some of the pathways related to cell division were also significantly over-represented, comprising for example “Mitotic M-M/G1 phases” or “Separation of sister chromatids” from the Reactome database, and “Aurora B signaling” or “PLK1 signaling events” from PID. Moreover, multiple protein kinases with known functionality in mitotic entry, spindle checkpoints, regulation of chromosome segregation and cytokinesis, such as PLK1 or NEK2, were more activated in the presence of VE-821 and both of them are known to be inhibited in an ATM-dependent manner in response to IR [27,28,29,30]. Notably, the regulation of PLK1 by ATR kinase after UV*-*but not gamma-irradiation has been very recently described [31].

In addition to these expected markers of G2/M checkpoint disruption, we further focused on the mechanisms involved in the G1/S transition and their possible modulation by ATR kinase. The direct involvement of ATR in regulation of transcription at G1/S transition after replication stress (*i.e.*, genotoxic stress in S-phase; caused by HU*-*treatment) has been reported recently [32]. Our results indicate the role of ATR in these processes after IR-induced DNA damage since a substantial portion of regulated phosphoproteins revealed in our study are involved in over-represented Reactome pathways such as: “Mitotic G1-G1/S phases”, “G1/S Transition”, “E2F mediated regulation of DNA replication”, and “Inhibition of replication initiation of damaged DNA by RB1/E2F1”.

The transcriptional regulators at G1/S comprise multiple proteins with different functions and thus are often grouped into four categories: activators (E2F1, E2F2, and E2F3), repressors (E2F4, E2F5, E2F6, E2F7, and E2F8), inhibitors (RB) and co-repressors (RBL1 and RBL2). Besides transcriptional regulation (e.g., E2F1 induces transcription of E2F6, E2F7, and E2F8 which in turn function as a negative feedback loop), phosphorylation mediated by CDK-Cyclin complexes, DNA repair kinases (ATM, ATR), and checkpoint kinases (CHK1 and CHK2) also have essential roles in regulation of G1/S [32]. Regarding the activators, cells exposed to IR in G1 phase were shown to accumulate E2F1 in an ATM-dependent manner (E2F1 stabilizing phosphorylation), which led to induction of S-phase and subsequently to apoptosis [33]. On the other hand, E2F6 repressor inhibition by ATR-CHK1-phosphorylation in response to replication stress (HU-treatment) resulted in pronounced E2F-dependent G1/S transcription, which in this particular scenario led to cell survival [32]. In summary, these two phosphorylation-mediated contradictory roles of the same transcriptional mechanism highlight the importance of phosphorylation in tight regulation of this process. Since we identified some significantly regulated phosphorylation sites of these proteins after ATR inhibition, we suggest that ATR kinase may contribute considerably to regulation of G1/S after gamma-irradiation at least in regards to HL-60 cells. Importantly, while the role of phosphorylation in modulation of pocket proteins (RB, RBL1, RBL2) activity is a well-described mechanism, the impact of posttranslational modifications on “atypical repressors” E2F7 and E2F8 functionality have not been described yet. We found two significantly increased phosphorylation sites in E2F8 and E2F4 repressors after ATR inhibition, however, further experiments are necessary to validate these phosphorylations as biologically relevant, which is our next goal.

Moreover, proteins involved in the pre-replicative complex (pre-RC) formation were significantly modified by phosphorylation after VE-821 treatment (e.g., “Activation of the pre-replicative complex” in over-represented Reactome pathways). Pre-RC formation and licensing, occurs in the G1 phase and the RC is activated at the G1/S transition by phosphorylation of its components, mainly ORCs (Origin recognition complex subunits) and MCMs (minichromosome maintenance complex subunits) complexes, CDC6, and CDT1, to initiate DNA-replication and thus progress into S phase [34]. We observed multiple phosphorylation sites with increased phosphorylation in ORCs and MCM proteins; most of them contained the SP/TP consensus motif typical for CDKs. Previously, ATR has been described to suppress origin firing in response to replication stress [9], which was confirmed here in the context of IR-induced DNA damage.

In conclusion, our analyses conducted in asynchronous p53 negative cells allowed us to observe a comprehensive impact of ATR kinase inhibition on cellular response to IR-induced genotoxic stress in all phases of the cell cycle. While the described work was conducted on a single cell line and the data need further validation, we found that ATR kinase inhibition modulated the mechanisms at the G1/S transition, impacted the intra-S-checkpoint, disrupted the G2/M checkpoint and altered the activity of kinases involved in mitosis.

## 4. Experimental Section

### 4.1. Cell Culture and Culture Conditions

HL-60 cells were obtained from the European Collection of Animal Cells Cultures (Porton Down, Salisbury, UK). Cells were cultured in Iscove’s modified Dulbecco’s media (IMDM) for SILAC containing 20% dialyzed fetal calf serum, 2 mM glutamine, 100 UI/mL penicillin, and 0.1 mg/mL streptomycin (all purchased from Sigma-Aldrich, St. Louis, MO, USA) at 37 °C, under controlled 5% CO_2_ and humidified atmosphere. Media were further supplemented with either unlabeled l-lysine (100 mg/L, K0) and l-arginine (84 mg/L, R0) or equimolar amounts of l-^13^C_6_-lysine (K6) and l-^13^C_6_-arginine (R6; isotopicaly labelled amino acids were purchased from Invitrogen, Carlsbad, CA, USA). l-proline (300 mg/L) was added to avoid metabolic conversion of arginine to proline [35]. For complete incorporation of labelled amino acids, cells were cultured for at least 6 doublings [36]. Cell counts were assessed by a hemocytometer and the cell membrane integrity was determined by the Trypan Blue exclusion technique (Sigma-Aldrich, St. Louis, MO, USA).

### 4.2. Gamma-Ray Irradiation

The selective inhibitor of ATR kinase, 3-amino-6-(4-(methylsulfonyl)phenyl)-*N*-phenylpyrazine-2-carboxamide (VE-821, APIs Chemical Co., Ltd. , Shanghai, China) was dissolved in DMSO in 10 mM aliquots and stored at −80 °C. Thirty min before irradiation, the inhibitor was added to the “heavy” cells (K6/R6) at concentration of 10 μM, the “light” cells (K0/R0) were treated with DMSO, whose final concentration in culture was lower than 0.1% to avoid the DMSO-induced differentiation of HL-60 cells. Both groups were irradiated by the dose of 6 Gy using a ^60^Co gamma-ray source (VF, Cerna Hora, Czech Republic) with a dose rate 0.5 Gy/min. After irradiation, the flasks were placed in an incubator. Three experimental replicates were analyzed.

### 4.3. Cell Lysis and Protein Digest

One hour after irradiation, the cells were washed with cold PBS and lysed as was published [37]:the cells were resuspended in ice-cold lysis buffer (50 mM ammonium bicarbonate, 1% sodium deoxycholate, phosphatase inhibitor cocktail 2). The lysate was immediately placed into boiling water bath and after 5 min incubation the samples were cooled to room temperature. To decrease viscosity, bensonase nuclease (2.5 U/μL) and MgCl_2_ (1.5 mM) were added to samples. The lysate was then clarified by centrifugation and the protein concentration was measured by bicinchoninic acid assay. Sample volumes corresponding to 1.75 mg of “light” proteins and 1.75 mg of “heavy” proteins were pooled together, reduced with dithiothreitol, alkylated with iodoacetamide and digested O/N with trypsin at an enzyme-to-substrate ratio of 1:60 (sequence grade modified trypsin, Promega Corporation, Madison, WI, USA). Sodium deoxycholate was then extracted by ethyl acetate [38] and peptides were desalted via 500 mg Supelco C18 SPE cartridges (Supelco Analyticals, Bellefonte, PA, USA). All other chemicals for cell lysis and protein digestion were purchased from Sigma-Aldrich.

### 4.4. Electrophoresis and Western Blotting

One hour after irradiation by 6 Gy, the HL-60 cells were washed with PBS and lysed. Whole cell extracts were prepared by lysis in 500 µL of lysis buffer (137 mM NaCl; 10% glycerol; 1% *n*-octyl-β-glucopyranoside; 50 mM NaF; 20 mM Tris, pH = 8; 1 mM Na_3_VO_4_; 1 tablet of protein inhibitors Complete™ Mini, Roche, Manheim, Germany). The lysates containing equal amount of protein (30 µg) were loaded onto a 12% SDS polyacrylamide gel. After electrophoresis, proteins were transferred to a polyvinylidene difluoride membrane (BioRad, Hercules, CA, USA), and hybridized with an appropriate antibody: anti-CHK1 and anti-CHK1 phosphorylated at serine 345 (1:500) from Cell Signaling (Danvers, MA, USA); anti-β-actin (1:2000) from Sigma. After washing, the blots were incubated with secondary peroxidase-conjugated antibody (Dako, Glostrup, Denmark) and the signal was developed with ECL detection kit (BM Chemiluminescence-POD, Roche) by exposure to a film.

### 4.5. Hydrophilic Interaction Liquid Chromatography Fractionation and Enrichment of Phosphopeptides

Dried peptide samples were fractionated by HILIC chromatography according to a protocol that has been published previously [39] using the 4.6 × 25 cm TSKgel^®^ (Tosoh Biosciences, Stuttgart, Germany) Amide-80 HR 5 μm particle column with the TSKgel^®^ Amide-80 HR 5 μm 4.6 × 1 cm guard column operated with Waters Separations Module 2695 at 0.5 mL/min. Briefly, 3.5 mg of evaporated samples were reconstituted in 80% B (98% acetonitrile (ACN)/0.1% trifluoroacetic acid (TFA), mobile phase A consisted of 2% acetonitrile with 0.1% TFA) and loaded onto the HILIC column. Peptides were then separated by a gradient of A over B from 80% to 60% B in 40 min and from 60% to 0% B in 5 min. Across the gradient, 22 fractions were collected (2 × 2 and 20 × 1 mL) from each replicate. Each fraction was then enriched for phosphopeptides using titanium dioxide chromatography [40]. At first, each fraction was supplemented with TFA and glutamic acid to reach final concentrations of 2% TFA and 100 mM glutamic acid. Titanium dioxide particles (Titansphere^®^ 5 μm particles, GL Sciences, Torrance, CA, USA) were suspended in the loading solution (65% acetonitrile, 2% TFA, 100 mM glutamic acid) and a particular volume of titanium dioxide suspension depending on an expected amount of peptides and phosphopeptides in a particular fraction was added to each sample microtube. Microparticles with bound phosphopeptides were then washed with 200 μL of loading solution, 200 μL of washing solution 1 (65% acetonitrile with 0.5% TFA), 200 μL of washing solution 2 (65% acetonitrile with 0.1% TFA) and 100 μL of washing solution 2. Phosphopetides were then eluted by 150 μL of elution solution (20% acetonitrile/NH_4_OH, pH 11.5) in two sequential elutions. Late fractions were subjected to the second enrichment. Eluates from the first and second enrichment were pooled together, acidified with 100% formic acid and placed in a SpeedVac until all crystals of ammonium formate were evaporated.

### 4.6. Mass Spectrometric Analysis

On-line LC-MS analyses were performed on Thermo Scientific Dionex Ultimate™ 3000 RSLCnano system (Thermo Scientific, Bremen, Germany) coupled through Nanospray Flex ion source with Q Exactive mass spectrometer (Thermo Scientific, Bremen, Germany). TiO_2_-enriched HILIC fractions were dissolved in 18 μL of 2% ACN/0.05% TFA and 2 μL were injected into RSLCnano system. Peptides were loaded on capillary trap column (C18 PepMap100, 3 µm, 100 A, 0.075 × 20 mm; Dionex) by 2% ACN/0.05% TFA mobile phase at flow rate 5 µL/min for 5 min and then eluted and separated on capillary column (C18 PepMap RSLC, 2 µm, 100 A, 0.075 × 150 mm; Dionex). Elution was carried out by step linear gradient of mobile phase B (80% ACN/0.1% FA) over mobile phase A (0.1% FA); from 4% to 36% B in 19 min and from 36% to 55% B in 6 min at flow rate 300 nL/min. Temperature of the column was 40 °C and eluent was monitored at 215 nm during the separation. Spraying voltage was 1.7 kV and heated capillary temperature was 220 °C. The mass spectrometer was operated in the positive ion mode performing survey MS (range 300 to 1800 *m*/*z*) and data-dependent MS/MS scans performed on the six most intense precursors with dynamic exclusion window of 40 s. MS scans were acquired with 70,000 resolution at 200 *m*/*z* from 1 × 10^6^ accumulated charges (maximum fill time was 100 ms). The lock mass at *m*/*z* 445.12003 ([(C_2_H_6_SiO)_6_ + H]^+^) was used for internal calibration of mass spectra. Intensity threshold for triggering MS/MS was set at 1 × 10^5^ for ions with *z* ≥ 2 with a 3 Da isolation window. Precursor ions were accumulated with AGC of 1 × 10^5^ (maximum fill time was 100 ms) and normalized collisional energy for HCD fragmentation was 27 units. MS/MS spectra were acquired with 17,500 resolution (at 200 *m*/*z*).

### 4.7. Data Processing

The raw files were processed with MaxQuant software version 1.3.0.5 [41]. Peak lists were searched against the human UniProt database (release February 2014) using Andromeda search engine [42]. Minimum peptide length was set to six amino acids and two missed cleavages were allowed. Carbamidomethylation of cysteines was set as fixed modification while oxidation of methionine, protein N-terminal acetylation, deamidation of glutamine and arginine and phosphorylation of serine, threonine, and tyrosine residues were used as variable modifications. Additionally, appropriate SILAC labels were selected (Arg6, Lys6). A mass tolerance of 6 and 20 ppm was allowed for MS and MS^2^ peaks, respectively. Only proteins, peptides and phosphorylation sites with FDR lower than 0.01 were accepted. For protein quantification only unmodified peptides, peptides oxidized at methionine residues, acetylated at *N*-terminus, or deamidated were accepted, both razor and unique peptides were used for the calculation of protein *H*/*L* ratios.

### 4.8. Bioinformatic Analysis

Contaminants and reversed hits were removed before further data processing and data were further manually inspected. The GRT was used to find differentially regulated phosphorylation sites. Phosphorylation sites quantified in all three replicates were included in GRT and the FDR was estimated non-parametrically as described by Zhou *et al*. [43]. The significance level of GRT was set to FDR < 0.01.

GO and signaling pathways over-representation analyses were performed using ConsensusPathDB, over-representation analysis web tool [14,15]. Only those proteins containing differentially regulated phosphorylation sites evaluated in GRT were included in the analyses. Significance was estimated using hypergeometric testing with Benjamini-Hochberg FDR correction (FDR < 0.01). All three GO classes (molecular function, cellular component and biological process) were statistically tested as well as the enrichment of proteins involved in signaling pathways stored in the Reactome pathway database [16] and in the PID [17]. The background reference set for the statistical analysis comprised all ConsensusPathDB entities: (i) annotated with a UniProt identifier that were included in at least one signaling pathway or (ii) that were annotated with a GO category which comprised at least one protein from the “regulated” dataset.

To analyze and visualize sequence motifs surrounding phosphorylation sites identified and quantified in our study, we employed the iceLogo tool and motif-x algorithm. In both motif analyses, the amino acid sequences (±7 residues) surrounding either significantly up*-* or down-regulated phosphorylation sites (determined using GRT) were tested against a background reference set composed of sequences surrounding all phosphorylation sites revealed in our study that reached the minimal localization probability 0.75 (*i.e.*, “class I phosphosites” [44]). In the iceLogo analysis the significance level was set to *p* < 0.01. Using motif-x, the significantly enriched linear motifs were extracted. Search parameters were set to at least 10 occurrences of a particular motif and the significance level of *p* < 0.00003 (which approximately corresponds to a *q*-value of 0.01 after Bonferroni correction for multiple hypothesis testing).

The changes in kinase activities were evaluated using three different tools for the predictions of phosphorylation site*-*specific kinases together with the evaluation of significance based on the Wilcoxon-Mann-Whitney test (“1D annotation enrichment” tool available in Perseus software, version 1.4.0.20 [45]). Input data were filtered to contain only those class I phosphorylation sites that were quantified in at least 2 of 3 replicates. In the first analysis, the consensus sequence motifs downloaded from the HPRD database were simply matched to sequences motifs of phosphorylation sites identified in our study (using “Add linear motifs” tool integrated in Perseus program). To further specify kinase predictions by combining motif scoring together with contextual motif (*i.e.*, protein-protein interaction scoring downloaded from the STRING database [46]) we employed two freely available predictors NetworKIN 3 [21] and iGPS [22]. Kinase-substrate relations predicted by NetworKIN 3 algorithm were further filtered according to the “NetworKIN score” (>2). For iGPS predictions, the “high” significance threshold was chosen (*i.e.*, FPR of 2% for S/T kinases and FPR of 4% for Y kinases). The significance threshold for the Wilcoxon-Mann-Whitney test was set to *p* < 0.05.

## 5. Conclusions

Defects in the DSB-induced DDR such as ATM and/or p53 deletion/mutation are common in human tumors, occur in up to 70% of cancer cells [47,48] and have been proposed to enable the proliferation of cancer cells with DNA lesions [49]. Our previous study suggested that in a p53-negative environment, the most effective inhibition involves ATR rather than ATM and/or DNA-PK [13]. This work provides for the first time a complex insight into the mechanism of radiosensitization of the p53-deficient model cell line (HL-60, human promyelocytic leukemia) by the highly specific ATR inhibitor, VE-821. We show here a powerful phosphoproteomic approach for investigation of the role of ATR-mediated signaling in the context of radiosensitization of cancer cells. We found that significantly regulated phosphorylation sites were involved in cellular processes related to mitosis, cell cycle regulation, and DNA repair. Furthermore, understanding these processes by applying the presented strategy might facilitate characterization of biomarkers for patients susceptible to selective ATR inhibition. Although the experiments presented here were performed only in one cell line, this study provides valuable information regarding complex ATR-mediated signaling changes, and should be further validated in other cell lines as well as in primary tumor samples from patients, which is our next goal. Our data underline the concept of radiosensitization in tumor cells as a promising new way to substantially increase efficacy of current cancer therapy.

## Supplementary Files

Supplementary File 1
